# The Differential Expression of miRNAs and a Preliminary Study on the Mechanism of miR-194-3p in Keloids

**DOI:** 10.1155/2019/8214923

**Published:** 2019-03-07

**Authors:** Zhishan Xu, Bingyu Guo, Peng Chang, Qiang Hui, Wei Li, Kai Tao

**Affiliations:** ^1^Department of Plastic and Reconstructive Surgery, The General Hospital of North Theater, PLA, Shenyang, China; ^2^The General Hospital of Shenyang Military Region, Postgraduate Training Base of Jinzhou Medical University, Shenyang, China

## Abstract

The aim of this study was to detect abnormally expressed microRNA (miRNA) in keloids and to study their functions. The differential expression of miRNAs in keloids and normal tissue was detected by gene microarray. MiRNA expression was verified by real-time PCR. A luciferase reporter gene assay, western blot, and real-time PCR were used to detect the effect of miR-194-3p on RUNX2. An MTT assay and a transwell assay were used to detect the effect of miR-194-3p in both primary cultured fibroblasts and HKF cells. Related proteins were analysed by western blot and real-time PCR. The expression of miR-194-3p was lower in keloids, and MiR-194-3p was shown to target RUNX2 directly. MiR-194-3p inhibited the proliferation and migration of fibroblasts through the inhibition of CDK4 and MMP2. MiR-194-3p and RUNX2 may become new targets for the prevention and treatment of keloids.

## 1. Introduction

The formation of a keloid seriously impacts the appearance of an individual; the symptoms vary to different degrees and can cause great harm to the physical and mental health of the patient [1]. The prevention and treatment of keloids have always been a research aim in the field of plastic surgery. Various factors, such as gene regulation, cytokines, genetics, and immunity, are known to be associated with the occurrence and development of keloids. However, research on the underlying mechanisms of these genes and cytokines is lacking [2, 3].

MiRNAs (MicroRNAs) are endogenous noncoding RNAs with lengths of approximately 18 to 22 nucleotides. Various physiological and pathological processes have been shown to be associated with the abnormal expression of miRNAs. Recent studies have demonstrated that many miRNAs may play pivotal roles in keloid scarring by regulating the proliferation, apoptosis, and metastasis of fibroblasts [4]. Studies have also shown that miRNAs such as miR-637, miR-34, and miR-31 can inhibit the development of keloids by down regulating the growth of fibroblasts [5–8].

We aimed to evaluate the expression levels of microRNAs in keloid fibroblasts and explore their regulatory roles in keloids. We hope that our research will provide new possibilities for the prevention and treatment of keloids.

## 2. Materials and Methods

### 2.1. Tissue Samples and Cell Sources

A total of 28 cases of keloid and corresponding normal skin tissue were derived from the General Hospital of North Theater. PLA between January 2015 and February 2017. All keloids were confirmed by clinical or pathological examination, the course of the disease was longer than 1 year, and patients had not received treatment. The specimens were removed and stored in liquid nitrogen. The experimental study was approved by the ethics committee of the General Hospital of Shenyang military region, and all patients were informed and provided consent.

HKF (human keloid fibroblast) cells obtained from the Shanghai cell bank of the Academy of Sciences were the primary culture of keloid fibroblasts. Cells were cultured in a 5% CO2 atmosphere at 37°C in DMEM containing 10% foetal bovine serum. The keloid tissue was minced and placed in pancreatic enzyme digestion at 37°C for 30 min. Then, DMEM containing 10% foetal bovine serum was added to the medium, terminating the reaction. Three to five generations of cells were used for these experiments.

### 2.2. RNA Extractions from Tissues

Total RNA was extracted from cells using the mirVana™ miRNA isolation kit (Ambion Life Technologies, Carlsbad, CA, USA), and RNA concentrations were measured using a NanoDrop® ND-1000 spectrophotometer (Thermo Fisher Scientific, Wilmington, DE, USA). RNA with an A260/A280 ratio of nearly 2.0 was considered to be high quality RNA.

### 2.3. Gene Chip Analysis

A microarray analysis was performed on RNA samples (3 paired) under microgravity and under static conditions in replicates. Expression data for each sample were obtained on the Affymetrix GeneChip Human Primeview Array. Hybridization was carried out for a duration of 16 h at 60 rpm at 48°C, and slides were scanned on the GeneChip microarray Scanner 3000 7G. Raw data were extracted after the slides were scanned, and raw datasets were analysed using GeneSpring GX 12.6 software, followed by differential gene expression (DE), fold-change, and cluster analysis.

### 2.4. Software Analysis

The miRDB database software was entered through a network search of miRDB (http://www.mirdb.org/) and targetscan (http://www.targetscan.org). Each target gene and its corresponding regulatory loci were obtained using the input target miRNA.

### 2.5. Real-Time PCR Reaction

RNA was reverse transcribed into cDNA according to the reverse transcription kit instructions. The reaction was carried out using the SYBR kit (Shenggong, Shanghai, China), and the reaction ran for 40 cycles. The expression of miR-194-3p was detected by stem-loop primers. The results were analysed with ABI Prism 7500 SDS software. The primers used are shown in Table 1. GAPDH and U6 were used for internal controls.

### 2.6. Dual Luciferase Reporter Gene Experiment

The primary cultures of keloid fibroblasts and HKF cells were divided into two groups. RUNX2 wild type/mutant type (wt/mut) and miR-194 mimic/control/antisense (AS) were transfected into cells. RUNX2 mut is a plasmid in which the binding site of RUNX2 to miR-194-3p is mutated. Twenty-four hours after the transfection, precooled lysate and the reaction solution were added. A luminescent meter was used to read the fluorescence value and analyse the data. The plasmids and RNA fragment we used were purchased from Shanghai Shenggong company (Shenggong, Shanghai, China).

### 2.7. Western Blot Analysis

Cells and tissues were lysed with radio immunoprecipitation assay (RIPA) buffer, mixed with Laemmli sample buffer (1×), and boiled. Proteins were subjected to 10% SDS-PAGE and electroblotted onto 0.22-*μ*M nitrocellulose membranes (BioRad Laboratories, USA). Membranes were blocked with Tris-buffered saline plus 0.2% Tween 20 (TBS-T) containing 3% BSA (Sigma Aldrich, USA), followed by overnight incubation with the primary antibody and washing with TBST buffer. Then, membranes were incubated with the secondary antibody (anti-mouse, HRP conjugate, 1:10000 Sigma Aldrich, USA), diluted in blocking buffer for 1 h at room temperature, and washed again with TBS-T. Antibody-reactive proteins were detected with enhanced chemiluminescence, Amersham ECL Plus western blotting detection reagents (GE health care, UK). RUNX2, CDK4, MMP2, and GAPDH antibodies were purchased from Santa Cruz Biotechnology (USA).

### 2.8. MTT Assays

The effect of miR-194 on cell proliferation was detected by MTT assay. The cells were inoculated in 96-well culture plates at a density of 1 × 10^4^ cells/well. After incubation for 24 h, the miR-194 mimic/control or miR-194 inhibitor/control was transfected into fibroblasts. Following the transfection, 5 mg/mL MTT was added at 0, 12, 24, 36, and 48 h. After 4 h of culture, the OD value at 490 nm was measured.

### 2.9. Transwell Assays

The effect of miR-194 on cell migration was detected by transwell assay. A total of 1 × 10^5^/ cells were inoculated in the upper chamber with 200 *μ*L of medium. A total of 600 *μ*L of medium were added to the lower chamber of the 24 wells. Twenty-four hours after the transfection, 95% ethanol was used to immobilize cells, and trypan blue was added. The number of cells was calculated by photographing and counting the cells under a microscope.

### 2.10. Construction of Stable Cell Lines

We constructed miR-194-3p overexpressing stable cell lines by transfecting cells with the miR-194-3p mimic and then selected with puromycin (1.5mg/ml). The miRNA mimic negative control was transfected as a control.

We constructed miR-194-3p low expression stable cell lines by transfecting cells with the miR-194-3p inhibitor and then selected with puromycin (1.5mg/ml). The miRNA inhibitor negative control was transfected as a control.

To stably silence RUNX2, cells were transfected with the si-RUNX2 fragment (Shanghai GeneChem Company), and a random fragment was used as a control. After 3 weeks, stable cells were selected, cultured, and amplified.

### 2.11. Wound Scratch Assays

Wound scratch assays were performed to detect cell migration. HKF cells were seeded in 6-well plates and allowed to reach confluence. An artificial wound was made using a 200 *μ*l pipette tip across the cell monolayer. Cells were rinsed with PBS and cultured in the medium. Wound closure was detected at 0 and 24 h and imaging performed under a microscope.

### 2.12. Statistical Methods

All experiments were repeated three times. SPSS, version 19 software, was used for statistical analysis, with data presented as x±s (standard deviation). Statistical significance was determined with unpaired Student t tests, except as noted for analyses of microarray data, which were examined with Fisher exact tests.* P* values less than 0.05 were considered statistically significant.

## 3. Result

### 3.1. Differential Expression of miRNA in Keloids

**Figure 1:**
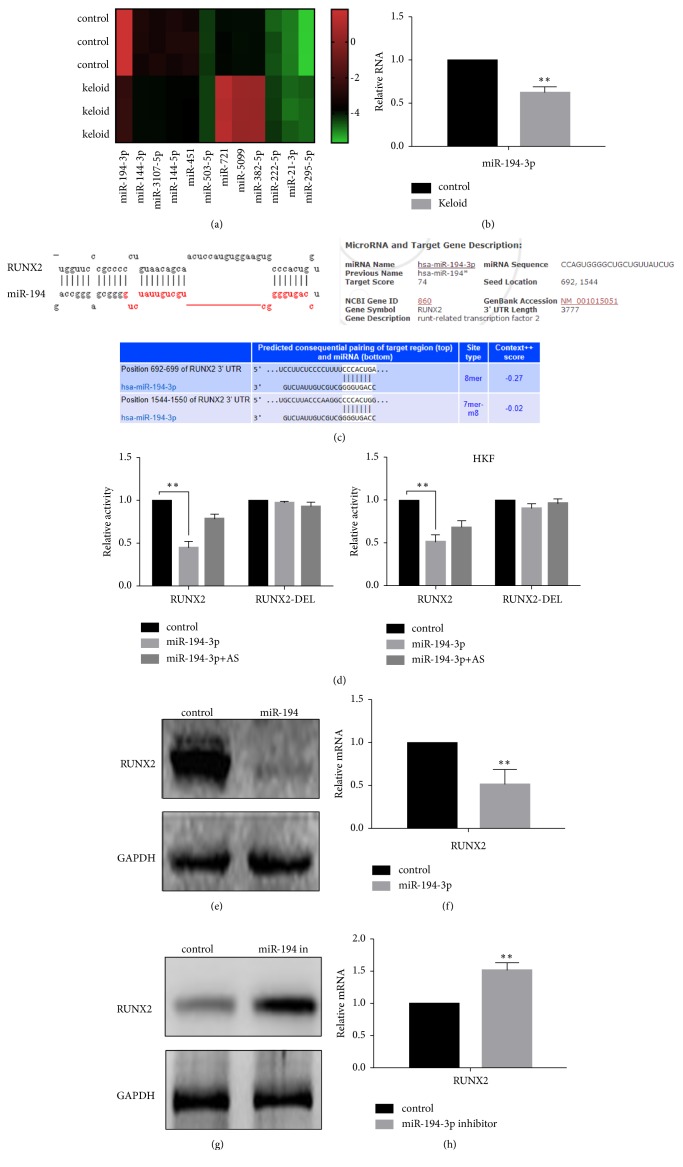
The expression of miRNA-194-3p is downregulated, and RUNX2 can be regulated. (a) Heat map of the microarray analysis. (b) As detected by real-time PCR, miR-194-3p has lower expression in keloids compared with normal skin, *∗∗ P*< 0.05. (c) The target binding site between miRNA-194-3p and RUNX2 was detected using miRDB and targetscan software. (d) The luciferase reporter gene experiment showed that miR-194-3p regulates the activity of RUNX2 in both primary cultured fibroblasts and HKF cells, *∗∗ P*< 0.05. (e) Western blot analysis showed that the expression of RUNX2 was inhibited after the overexpression of miR-194-3p. (2C) Real-time PCR showed that the expression of RUNX2 mRNA was inhibited after the overexpression of miR-194-3p. (f) The expression level of RUNX2 increased after the inhibition of miR-194-3p. (g) When miR-194-3p was suppressed, the level of RUNX2 mRNA increased.

The results of the chip detection revealed that many miRNAs, including miR-194-3p, miR-451, and miR-144-5p, were differentially expressed in keloids compared with adjacent tissue (Figure 1(a)). These results are shown in Table 2.

### 3.2. The Expression of miRNA-194-3p Is Downregulated, and RUNX2 Can Be Regulated

All 28 cases of keloid tissue we collected were tested by real-time PCR. The results showed that miR-194-3p has lower expression in keloid tissue (Table 3, Figure 1(b)). Using miRDB software, we found that miRNA-194-3p has a binding site in the 3′ UTR region of RUNX2 (Figure 1(c)). To determine the regulatory effect of miR-194-3p on RUNX2, we conducted a reporter gene experiment using HKF cells and primary cultured cells. The results indicated that miR-194-3p significantly inhibited the activity of RUNX2. After transfection with miR-194-3p, the activity of RUNX2 was significantly lower than that of the control group (unintentional chain control). The results also showed that when miRNA-194-3p and its antisense chain (AS) or RUNX2 DEL were cotransfected, the inhibitory effect of miRNA-194-3p on RUNX2 activity almost disappeared (Figure 1(d)). We transfected the miR-194-3p mimic or the miR-194-3p inhibitor into HKF cells and found that miR-194-3p significantly inhibited the expression of RUNX2. In contrast, when miR-194-3p was inhibited, the expression of RUNX2 was elevated (Figures 1(e)–1(h)).

### 3.3. The Effect of miR-194-3p on the Proliferation of Fibroblasts

**Figure 2:**
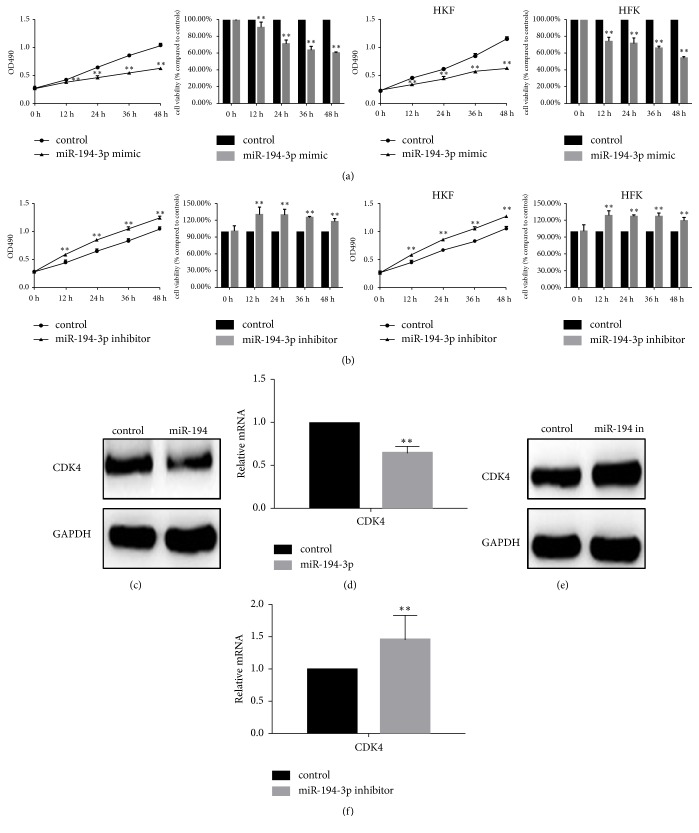
Effect of miR-194-3p on the proliferation of primary cultured fibroblasts and HKF cells. (a, b) The effect of miR-194-3p on cell proliferation was detected by MTT assay, and there was a significant difference between groups, *∗∗ P*< 0.05. (c) Western blot analysis revealed that the expression of CDK4 was inhibited after the overexpression of miR-194-3p. (d) Real-time PCR showed that the mRNA expression of CDK4 was suppressed after miR-194-3p overexpression, *∗∗ P*< 0.05. (e) When miR-194-3p was inhibited, the expression of CDK4 increased. (f) When miR-194-3p was inhibited, mRNA expression of CDK4 was elevated, *∗∗ P*< 0.05.

After transfecting the miR-194-3p mimic or inhibitor into cells, the MTT assay revealed that miR-194-3p significantly inhibited the proliferation of cells. When miR-194-3p was inhibited, the proliferation of cells increased significantly (Figures 2(a) and 2(b)).

CDK4 is a cell cycle-related protein downstream of the RUNX2 pathway [9]. Through western blot analysis and real-time PCR, it was determined that miR-194-3p significantly inhibited the expression of CDK4 (Figures 2(c)–2(f)).

### 3.4. The Effect of miR-194-3p on the Migration of Fibroblasts

**Figure 3:**
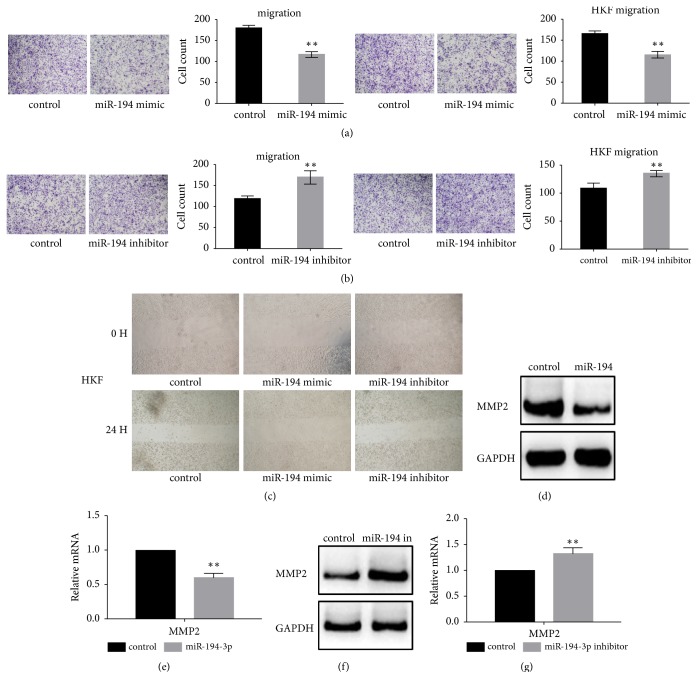
Effect of miR-194-3p on the migration of fibroblasts. (a) When miR-194-3p was overexpressed, the migration of fibroblasts and HKF cells decreased, *∗∗ P*< 0.05. (b) When miR-194-3p was inhibited, the migration of fibroblasts and HKF was observed, *∗∗ P*< 0.05. (c) Wound scratch assay was performed to analyze cell migration. miR-194-3p overexpression inhibited HKF migration, while miR-194-3p inhibitor promoted HKF migration. (d, e) Western blot analysis and real-time PCR showed that the expression of MMP2 was inhibited after miR-194-3p was overexpressed, *∗∗ P*< 0.05. (f, g) Western blot analysis and real-time PCR showed that the expression of MMP2 increased after the inhibition of miR-194-3p, *∗∗ P*< 0.05.

The results of the transwell assay showed that the overexpression of miR-194-3p significantly inhibited the migration of cells (Figure 3(a)). When miR-194-3p was inhibited, the migration of cells increased significantly (Figure 3(b)). Similar results were obtained in wound scratch (Figure 3(c)). Western blot analysis and real-time PCR revealed that the overexpression of miR-194-3p significantly inhibited the expression of MMP2 (Figures 3(d)–3(g)). MMP2, similar to CDK4, is a known target gene in the RUNX2 signalling pathway [10, 11]. We conclude that the inhibitory effect of miR-194-3p on MMP2 may reverse the inhibition of RUNX2 by miR-194-3p.

### 3.5. RUNX2 Is Crucial to Cell Proliferation and the Migration of HKF Cells

**Figure 4:**
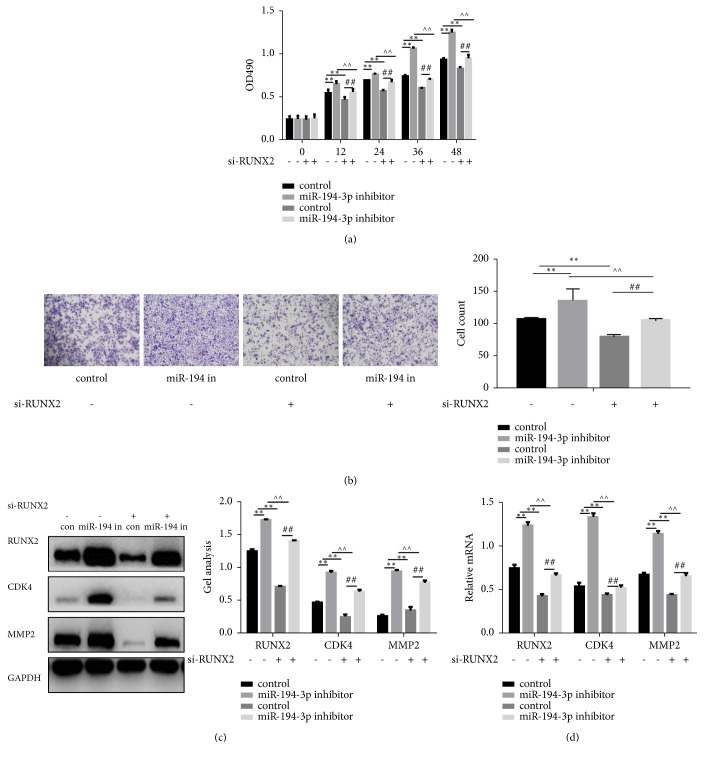
RUNX2 is crucial to the proliferation and migration of HKF cells (a) Effect of miR-194-3p siRNA and RUNX2 siRNA on cell proliferation as measured by MTT assay. Data are presented as the mean ± SEM. *∗∗ P*< 0.05 vs. miR-194-3p inhibitor group, *∗∗ P*< 0.05 vs. si-RUNX2 group, ∧∧* P*< 0.05 miR-194-3p inhibitor group vs. si-RUNX2+miR-194-3p inhibitor group, and* ## P*< 0.05 si-RUNX2 group vs. si-RUNX2+miR-194-3p inhibitor group. (b) Effect of miR-194-3p siRNA and RUNX2 siRNA on cell migration ability, detected with the transwell assay. Cells were counted, and results represent the mean ± SD of three experiments. ×200 *∗∗ P*< 0.05 vs. miR-194-3p inhibitor group, *∗∗ P*< 0.05 vs. si-RUNX2 group, ∧∧* P*< 0.05 miR-194-3p inhibitor group vs. si-RUNX2+miR-194-3p inhibitor group, and* ## P*< 0.05 si-RUNX2 group vs. si-RUNX2+miR-194-3p inhibitor group. (c, d) The effects of miR-194-3p siRNA and RUNX2 siRNA on RUNX2, Cyclin D1, and MMP2 were detected by western blotting and real-time PCR. Data are shown as the mean ± SEM. *∗∗ P*< 0.05 vs. miR-194-3p inhibitor group, *∗∗ P*< 0.05 vs. si-RUNX2 group, ∧∧* P*< 0.05 miR-194-3p inhibitor group vs. si-RUNX2+miR-194-3p inhibitor group, and* ## P*< 0.05 si-RUNX2 group vs. si-RUNX2+miR-194-3p inhibitor group.

The MTT and transwell assays demonstrated that RUNX2 small interfering (si)RNA significantly suppressed cell proliferation and cell migration (*P*<0.05) (Figures 4(a) and 2(b)). The inhibition of si-RUNX2 was blocked by the miR-194-3p inhibitor. Western blot analysis and real-time PCR showed that cotreatment with RUNX2 siRNA and the miR-194-3p inhibitor in cells leads to significant upregulation of RUNX2, CDK4, and MMP2 (*P*<0.05) (Figures 4(c) and 4(d)). These results suggest that miR-194-3p may regulate the proliferation and metastasis of HKF cells via the RUNX2 signalling pathway.

## 4. Discussion

miRNAs are a class of endogenous noncoding small RNA molecules that have regulatory and control functions on genes. Such noncoding small RNA molecules play an important role in processes including biological development, metabolism, differentiation, proliferation, and apoptosis. Previous studies have indicated that abnormal expression of miRNAs can cause disease and even cancer, as certain miRNAs function similarly to tumour suppressor genes [12, 13]. Keloids are a type of solid tumour and, in recent years, researchers have focused on the effect of miRNAs on keloid development. Research into the role of miRNAs on keloids is expected to provide a new avenue for the treatment of tumour-like keloid pathologic tissue.

It was shown that miRNAs are involved in the regulation of fibroblast proliferation, differentiation, and extracellular matrix synthesis. In addition, a complex functional network is formed between miRNA and keloids [14, 15]. It has been reported that miRNA-199a-5p has lower expression in keloids, which may be related to the arrest of S phase or G2/M phase [7]. MiR-205-5p inhibits the expression of keloid fibroblasts by inhibiting the VEGF pathway [16]. Other studies have determined that the expression level of miR-29a/b/c in keloid fibroblasts is significantly decreased. The lower expression levels of miR-29a and miR-196a increases the secretion of type I/III collagen in fibroblasts, which aggravates the formation of keloids [17, 18]. MiR-21 has higher expression levels in keloid fibroblasts, which can enhance cell proliferation through TGF-beta signalling. Another region of miR-21 can be combined with Smad7 mRNA in the 3′ UTR of the target gene, Smad7, reducing the expression of this gene and leading to increased generation of fibroblast extracellular matrix [19]. It has been found that miR-194 has relatively low expression in many types of tumours and regulates many biological functions of cells. MiR-194 can inhibit apoptosis, proliferation, and metastasis of hepatoma and osteosarcoma cells [20, 21]. Studies have also shown that the expression of miR-194 is associated with the progression of gastric cancer [22]. However, there has been no report on the expression and function of miR-194 in keloids. In our study, we first explored the mechanism of miR-194-3p in keloids.

We found that there are many differentially expressed miRNAs in keloids compared with adjacent tissues. Among these differentially expressed miRNAs, we showed for the first time that the expression of miR-194-3p in keloids was significantly lower compared with normal tissue. Real-time PCR revealed that miR-194-3p was downregulated in keloid tissues. The lower miR-194-3p expression was significantly correlated with family genetic history. However, due to the difficulty of sample collection, the sample size of this study is relatively small, which is a limitation of our study. To study the effect of miR-194-3p on keloids, miRDB and targetscan software were used to predict the target genes of miR-194-3p. Further analysis revealed that the 3′ UTR region of miRNA-194-3p has a binding site leading to the direct regulation of the expression of RUNX2.

RUNX2 is a major transcription factor regulating organ formation, and it affects the proliferation and migration of many solid tumours [23]. RUNX2 was shown to be an inducer of aortic fibrosis and stiffness which could increase migratory ability in HKF [24]. However, there are few reports on the role of RUNX2 in keloids. We determined that miRNA-194-3p inhibits RUNX2 in cells.

Studies showed that downregulation of RUNX2 expression significantly inhibited cell proliferation, migration, and invasion of cells [25]. We found that miR-194-3p may inhibit the growth and migration of fibroblasts by inhibiting RUNX2. MTT assay revealed that miR-194-3p significantly inhibited the proliferation of cells. We further showed that the inhibitory effect of miR-194-3p on cell proliferation may be achieved through the inhibition of CDK4.

Studies have shown that RUNX2 can improve the migratory ability of various cells [26]. The transwell assay was used to detect the migration of fibroblasts, and the results showed that miR-194-3p significantly inhibited cell migration. The secretion of MMP2 can effectively degrade the basement membrane and create conditions that promote cell migration. Based on our study, we hypothesize that the inhibitory effect of miR-194-3p on cell migration may be achieved through the inhibition of MMP2.

In conclusion, our study revealed that miR-194-3p significantly inhibits the proliferation and migration of fibroblasts. The inhibitory effect of miR-194-3p on fibroblasts may be achieved through the targeting effect of miR-194-3p on RUNX2. MiR-194-3p may become a potential target for the prevention and treatment of keloids.

## Figures and Tables

**Table 1:** Real time PCR primer sequences.

Name	Forward primer	Reverse primer
miR-194-3p	ACACTCCCAGUGGGGCUG	CAGAUAACAGTTGAGAGTACAT
U6	CTCGCTTCGGCAGCACA	ACGCTTCACGAATTTGCGT
RUNX2	CCGCCTCAGTGATTTAGGGC	GGGTCTGTAATCTGACTCTGTCC
CDK4	GGGCTTTGAGGCTGTCTACC	GTCCACGCTGGCATCTTCTG
MMP2	CTACCTCTCGAATGAGCCAG	CACTCCGGATTACCTTCAT
GAPDH	CATCCCTTCTCCCCACACAC	AGTCCCAGGGCTTTGATTTG

**Table 2:** Differential expression of miRNAs in keloids.

MiRNA	*P* values	Fold change	Trend
Hsa-miR-194-3p	1.21E-05	65.283	down
Hsa-miR-144-3p	0.010	3.422	down
Hsa-miR-3107-5p	0.003	4.061	down
Hsa-miR-144-5p	0.011	3.287	down
Hsa-miR-451	0.007	3.198	down
Hsa-miR-503-5p	0.004	2.983	down
Hsa-miR-721	7.47E-06	42.093	up
Hsa-miR-5099	0.001	38.392	up
Hsa-miR-382-5p	0.001	24.321	up
Hsa-miR-222-5p	0.003	3.982	up
Hsa-miR-21-3p	0.001	2.332	up
Hsa-miR-295-5p	0.027	1.873	up

**Table 3:** The relationship between miR-194-3p and keloid.

Variables	Description	No. of patient	miR-194-3p expression		*χ* ^2^	*P* value
			Low	High		
Gender	Male	13	9	4	0.021	0.885
	Female	15	10	5		

Age(years)	<40	17	13	4	2.391	0.122
	≥40	11	5	6		

Family heredity	Yes	15	13	2	0.524	0.022*∗*
	no	13	6	7		

position	Chest	7	4	3	0.3889	0.943
	shoulder	8	5	3		
	Ear	9	6	3		
	other	4	3	1		

Pathogeny	trauma	12	5	9	0.124	0.940
	Operation	10	9	3		
	other	6	4	0		

## Data Availability

The data used to support the findings of this study are included within the article
